# Recent Progress in Studies of Arterivirus- and Coronavirus-Host Interactions

**DOI:** 10.3390/v4060980

**Published:** 2012-06-19

**Authors:** Yanxin Zhong, Yong Wah Tan, Ding Xiang Liu

**Affiliations:** School of Biological Sciences, Nanyang Technological Sciences, 60 Nanyang Drive, Singapore 637551, Singapore; Email: ZH0007IN@ntu.edu.sg (Y.Z.); g0600216@nus.edu.sg (Y.W.T.)

**Keywords:** virus-host interactions, coronavirus, arterivirus

## Abstract

Animal coronaviruses, such as infectious bronchitis virus (IBV), and arteriviruses, such as porcine reproductive and respiratory syndrome virus (PRRSV), are able to manifest highly contagious infections in their specific native hosts, thereby arising in critical economic damage to animal industries. This review discusses recent progress in studies of virus-host interactions during animal and human coronavirus and arterivirus infections, with emphasis on IBV-host cell interactions. These interactions may be directly involved in viral replication or lead to the alteration of certain signaling pathways, such as cell stress response and innate immunity, to facilitate viral replication and pathogenesis.

## 1. Introduction

Coronaviruses, together with arteriviruses and toroviruses, belong in the order *Nidovirales*, a group of large, non-segmented, positive sense and single stranded RNA animal viruses that produce an extensive 3'-nested set of subgenomic mRNAs for transcription during infection [1] (Table 1). Nidoviruses such as avian infectious bronchitis coronavirus (IBV), human coronavirus 229E (HCoV-229E), equine arteritis virus (EAV) and the porcine reproductive and respiratory syndrome arterivirus (PRRSV) are important pathogens of both human and animals [2,3,4], and are commonly associated with mild respiratory and enteric diseases, although they are also known to cause more critical lower respiratory tract illness, such as the severe acute respiratory syndrome coronavirus (SARS-CoV) epidemic that occurred in 2003 [5].

As these viruses infect livestock, coronaviral and arteriviral infections in farms have resulted in large-scale economic losses in farming nations, and are therefore of exceptional veterinary research value. Coronaviruses in fowls, as exemplified by the highly contagious IBV in chickens, can be highly lethal to young chicks. IBV is the etiological agent of infectious bronchitis, an avian disease that is mainly associated with upper respiratory and urogenital tract infections in adult chickens, and to a lesser extent, nephrogenic infections, or inflammation of the kidneys [6,7]. The impact of IBV infection is also emphatically increased as a consequence of its enhancement of diseases associated with lethal co-infections by bacteria and mycoplasmas [8,9]. Domestic animals such as cats and dogs are also susceptible to coronaviruses. Feline coronaviruses, especially feline infectious peritonitis virus (FIPV)—a mutation of Feline Enteric Coronavirus (FECV), may induce lethal diseases in cats [10], while canine coronavirus (CCoV) infections, which cause canine enteric illness in dogs, are prevalent as well [11]. In larger livestock like pigs and cattle, on the other hand, coronaviruses and arteriviruses typically establish enteric infections. An infection or outbreak can cause severe economic losses from the death of young offspring, lifelong impact on the yield of animal produce such as eggs and milk, weight losses and the general health of the population. Bovine coronavirus (BCV), for example, causes Winter Dysentery (WD) in adult cows and diarrhea in young calves [12].

The pathogenicity of these viruses is typically species-dependent, as is the severity of infection; they infect mainly their natural hosts and/or species that are closely related. Certain virus infections, however, can cross the species barrier, with the prime example being the zoonotic SARS-CoV, a novel coronavirus that is thought to have originated from bats before it adapted to its intermediate host, civet cats, and finally to humans [13]. Bat colonies, which are scattered worldwide, are widely known to play host to a variety of coronaviral and adenoviral pathogens while acting as natural wildlife reservoirs of these viruses [14,15,16].

Coronavirus infections are also generally tissue-specific—the Transmissible Gastroenteritis Coronavirus (TGEV), for example, affects mainly the gastrointestinal tract [17] that may lead to the onset of fatal watery diarrhoea and severe dehydration in pigs [18], while human coronaviruses mostly cause respiratory infections [4].

With respect to their significance to the economy, vaccines have also been developed for many of these viruses in a bid to prevent localized infections from progressing into serious outbreaks. This has, however, proven to be a hard battle as the vaccines are unable to provide complete cross-protection among the various serotypes of each virus [19].

**Table 1:** Classification of nidoviruses.

Order	Family	Sub-Family	Genera	Representative Animal Species	Host and Tissue Tropism
**Nidovirales**	Coronaviridae	Coronavirinae	Alphacoronavirus	Transmissible gastroenteritis virus	Pigs (GI)
				Feline Coronavirus	Domestic cats (GI, Peritoneal)
				Bovine Coronavirus	Cattle (GI)
				Bat Coronavirus HKU2 and HKU8	Bats (Carrier)
			Betacoronavirus	Mouse Hepatitis Virus	Mice (Res, GI, Hep, CNS)
				Bat Coronavirus HKU9	Bats (Carrier)
				Severe Acute Respiratory Syndrome Coronavrus^#^	Palm Civets (Carrier)Bats (Carrier)
			Gammacoronavirus	Avian Infectious Bronchitis Virus	Chickens (Res, Neph, Rep)
				SW1 virus	Beluga Whale (Res, Hep)
		Torovirinae	Bafinivirus	White bream virus	Fish (GI, Hep)
			Torovirus	Breda virus (bovine torovirus)	Cattle (GI)
	Arteriviridae	-	Arterivirius	Porcine reproductive and respiratory syndrome virus	Pigs (Resp, Rep)
				Equine arteritis virus	Horses (Resp, Rep)
	Roniviridae	-	Okavirus	Yellow head virus	Crustaceans (prawns); cephalothorax

#: Same as the virus isolated in humans. (Abbreviations) GI: Gastrointestinal System, Resp: Respiratory System, Rep: Reproductive System, CNS: Central Nervous System, Neph: Nephrogenic System, Hep: Hepatic System.

### 1.1. Virus Infection and Host Responses

During infection, the virus replicates in the host cytosol amidst a myriad of host signaling pathways and systems such that interaction between the virus and the host systems is inevitable. Virus infection and the consequent host cell response also involve complicated interaction between various host cellular and viral networks. Virus-host interplay occurs at multiple points during the virus replication cycle, from entry to exit. The nature of such interactions can range from a simple exploitation of existing host machinery to destructive interactions that modulate the host environment to the advantage of the virus while inhibiting host activities. One of the most important interactions between virus and host is the modulation of host cell environment, such that the latter is converted into one in which the virus can replicate successfully. Viruses also regulate the differential expression of host genes, as well as various host antiviral defense mechanisms, for more efficient replication. 

Previous studies on the infection of different hosts by nidoviruses, for example, have shown various modifications in host innate immune and stress responses, cell cycle, autophagy and cell death pathways [20,21,22,23], all of which will be discussed in this review. The significance of host components being used to supplement the gene-poor virus in various processes cannot be dismissed, for although they typically serve as enhancers, they could also become major pathogenicity factors.

## 2. The Effect of Virus Infection on Apoptosis

A number of stimuli can precipitate apoptotic events, including cell homeostatic imbalance such as cell stress, and the binding of ligands to cell surface “death” receptors; these in turn trigger the onset of major apoptotic pathways: the extrinsic or intrinsic pathway [24]. 

### 2.1. Extrinsic and Intrinsic Apoptotic Pathways

The extrinsic pathway can be induced by several cytokine “death” receptors from the tumor necrosis factor (TNF) family, such as Fas (Apo1/CD95) [25]. Upon recruitment of their respective ligands, they form complexes that subsequently bind death effector domain (DED)-containing pro‑Cysteine Aspartyl-Specific Proteases (pro-caspases), in particular pro-caspase-8, where the activation and consequent oligomerization of which further serves is a signal for downstream activations, thus pledging the doomed cell towards its own death [26]. The intrinsic pathway, on the other hand, is activated by the release of cytochrome *c* from the mitochondria into the cytoplasm [27]. In the cytosol, cytochrome *c* binds the apoptotic protease-activating factor (Apaf1); together, they form an apoptosome that leads to the release of active caspase 9. 

Apoptotic mitochondrial events are also regulated primarily through the activation of pro-survival and pro-apoptotic proteins [28]. The Bcl-2 family of proteins constitutes a critical control point in the regulation of apoptosis. They form three major protein subgroups: the Bcl-2 homology (BH) 3, or BH3-only proteins [e.g., BH3-interacting domain death agonist (Bid), BCL2-associated agonist of cell death (Bad)], Bax-like proteins [e.g., BCL-2-antagonist/killer 1 (Bak), BCL-2-associated X (Bax)] and the Bcl-2-like factors [e.g., Myeloid cell leukemia-1 (Mcl-1), Bcl-extra large (Bcl-X_L_)] [29]. BH3-only and Bax-like proteins are essential initiators of apoptosis while the Bcl-2-like proteins are pro-survival factors that safeguard the cells against apoptosis.

Both caspase 8, from the extrinsic pathway, and caspase 9, from the intrinsic pathway, have been observed to activate the main effector caspase 3, which in turn activates a caspase cascade to eventually evoke the morphological hallmarks of apoptosis such as DNA fragmentation [30,31]. 

### 2.2. Other Apoptotic Pathways

A third apoptotic pathway, induced by prolonged endoplasmic reticulum (ER) stress, has also been shown to activate multiple downstream apoptotic targets, including rodent caspase 12, growth arrest and DNA damage-inducible gene 153 (GADD153), also known as the transcription factor C/EBP homologous protein (CHOP) as well as activation of the pro-apoptotic c-Jun NH2-terminal kinase (JNK) [32]. Human pro-inflammatory caspase 4, a nearly identical paralogue of the rodent form of caspase 12, has also been shown to possess comparable roles in ER-stressed apoptosis [33]. 

JNK activation is mediated by ER transmembrane protein kinases, while CHOP is triggered by ER stress at the transcriptional level [32]. The downstream apoptotic activities of both JNK and CHOP have also been postulated, at least in part, to be connected with the Bcl-2 family of proteins (Bak and Bax) for recruitment to the ER and subsequent initiation of apoptosis in response to stress [32]. 

### 2.3. Viruses and Apoptosis

As viruses depend on the host cells they infect in order to reproduce, apoptosis is often employed as an important host antiviral defense mechanism that, as a protective measure, leads to the abortion of virus infection such that viral productivity and persistent infectivity is consequently limited [34]. In many cases, p53 and the Bcl-2 family of proteins have been shown to be the main mediators that induce the beleaguered cell to undergo self-induced death at various stages of the infection cycle [35]. 

Moreover, host endosomal membranes are forced to undergo conformational changes for the fusion of virus and host cell membranes during virus uncoating; membrane integrity is also antagonized during the process of virus disassembly. As such, these drastic alterations to membranes may elicit downstream pro-death signals that prompt infected cells to commit suicide [36].

However, certain viruses have evolved strategies to both counteract and induce apoptosis in order to maximize the production of virus progeny and promote its spread to neighbouring cells. An increasing number of known viruses from different families, including arteriviruses, have been found to induce apoptosis during their infection cycle, which may possibly contribute to the cytotoxicity associated with virus infections, especially during late stages of infection [37]. Membrane-bound cell fragments are also produced as apoptotic bodies to be phagocytosed by surrounding cells. This provides an excellent method for a virus to disperse its progeny without eliciting host immune response [38]. 

While more comprehensive work needs to be done to paint a clearer picture of how coronaviruses and arteriviruses regulate apoptosis during infection, recent reports have suggested the possible activation of more than one apoptotic pathway during infection. EAV, an arterivirus that is prevalent among global horse populations and which may induce abortions in pregnant mares [39], has been shown to activate apoptosis through the initiation of caspase-8-dependent mechanisms, which is followed by mitochondria-dependent caspase-9 activation mechanisms [40]. PPRSV, a virus that causes respiratory tract problems in young pigs and has a commercially significant impact on swine industries worldwide as a result of reproductive impairment in breeding livestock [41], has also been implicated to regulate both apoptosis and necrosis during infection [42,43]. Replication of TGEV in porcine kidney cells, as well as that of canine coronavirus type II (CCoV-II) in canine fibrosarcoma cells, on the other hand, has been reported to induce apoptosis both through Fas/FasL-activation and mitochondrial-dependent pathways [44,45]. 

**Figure 1:**
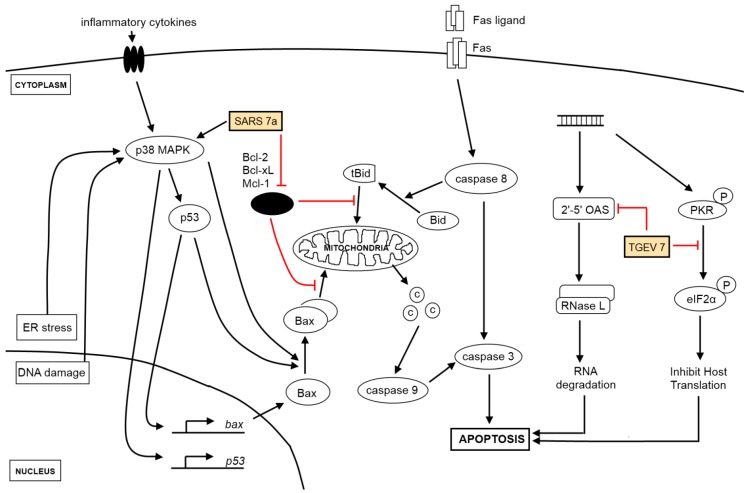
Viral genes and the activation of apoptosis. Extrinsic signals from receptors (e.g., Fas ligand) culminate in the activation of caspase 8, which activates the effector caspase 3 while intrinsic signaling requires the participation of the mitochondria in releasing cytochrome *c* (shown as circles labeled “C”) to activate caspase 9 for the downstream activation of caspase 3. Key proteins in the intrinsic apoptosis signaling pathway are p53, both pro-apoptotic (e.g., Bax, Bak) and anti-apoptotic proteins (e.g., Bcl-2, Bcl-_X_L) from the Bcl-2 family. Viral genes (see yellow boxes), which act at multiple points along the different signaling pathways, target both the extrinsic and intrinsic apoptosis signaling pathways and enhances the pro-apoptotic effect brought upon by virus infection. Anti-apoptotic proteins (black oval) listed are key anti-apoptotic members from the Bcl-2 family of proteins. Other pathways triggered by coronavirus infections, such as ER stress and DNA damage response, activate apoptotic signaling pathways as well.

### 2.5. IBV-Induced Apoptosis and Its Regulation

Although IBV is an avian virus, it can acclimate to primate cells and has been demonstrated to conquer the host species barrier and become zoonotic to infect both human and animal cells [46,47]. IBV also triggers apoptosis during the late stages of its cytolytic infection cycle. Specifically, IBV‑induced apoptosis has been shown to involve Bcl-2 family of proteins [48], through caspase‑dependent [49] and p53-independent [50] pathways in cultured mammalian cells. The modulation of Bcl-2 family proteins during IBV infection has also been postulated to be under the regulation of signaling pathways such as ER stress and Mitogen-Activated Protein Kinase/Extracellular signal-Regulated Kinase (MAPK/ERK) pathways [48]. The effects of these pathways on the regulation of other host defenses will be discussed in later paragraphs.

### 2.6. Viral Genes Implicated in Apoptosis

While viral genes can also manipulate the induction of apoptosis to the benefit of viruses, only two have been reported in the case of coronaviruses. The unique SARS-CoV encoded protein, 7a, was discovered to have caspase-dependent, pro-apoptotic functions and may function as a plausible source of virus-derived apoptotic signal [51], while TGEV accessory gene 7, present only in coronaviruses classified under genus α1 [52], thwarts virulence and host-induced antiviral mechanisms through the negative modulation of downstream caspase-dependent apoptotic pathways [53] (Figure 1). 

## 3. The Effect of Virus Infection on Host Innate Immunity

The maintenance of apoptosis is also important in the establishment and governance of immune responses in a cell. A loss in the control of apoptosis leads to an imbalance in cell homeostasis, which ultimately affects immune sensitivity [54]. The presence of apoptotic cells, particularly in existence with infectious agents, may also lead to the mobilization and initiation of innate immune defenses [55]. This crosstalk between apoptosis and innate immunity is therefore of considerable importance during pathogenic infection and can be manipulated by both host and pathogen, either as a form of immune defense or immune evasion, respectively [56].

### 3.1. Pathogen Detection and Host Antiviral Defense

When the host immune system is exposed to viral pathogens, it reacts straightaway by triggering a diverse array of defense mechanisms in order to establish a more efficacious shield. The first line of defense is the mounting of an innate immune response, as characterized by the increased production of type I interferons (IFN-α and IFN-β) and other inflammatory cytokines. These, in turn, choreograph the expression of downstream IFN-stimulated genes (ISGs) and activate several signaling pathways, all of which collaboratively lead to the induction of a protective antiviral state and, subsequently, the inhibition of both viral replication and proliferation [57]. 

### 3.2. Interferons and the Antiviral Response

The cytokine family of interferons is dedicated to the conveyance of the presence of infection, as well as the expedition of numerous connections among the cells that provide protection against, or eradication of, foreign pathogens. Other than interfering with viral progeny production in host cells—hence the name ‘interferon’—IFNs also induce Natural Killer (NK) cells and macrophages to ‘kill’ or engulf infected cells, increase antigen presentation to thymus (T) cell lymphocytes for rapid recognition of infected cells and bring about virus resistance to new uninfected cells [58]. IFNs are conventionally classified into three types: Type I (IFN-α, IFN-β and IFN-ω), Type II (IFN-γ) and the more recently identified Type III (IFN-λ1, IFN-λ2 and IFN-λ3) [59,60]. Over time, mammalian hosts have gradually developed a multitude of cellular sensors for the detection of viral infection, and it is the involvement and operation of these cellular protein receptors that eventually leads, through an intricate network of pathways, to the expression of type 1 IFNs. Major receptor systems that conduct immune surveillance and trigger the production and subsequent release of type I IFNs are known as pattern recognition receptors (PPRs), which detect viral infection through the identification of various pathogen-associated molecular patterns (PAMPs); PPR families include the toll-like receptor (TLR), RIG-like helicase (RLH) and Nucleotide-binding oligomerization domain (NOD)-like receptor (NLRs) families [61]. 

### 3.3. Pattern Recognition Receptor Families

The TLR family is mainly made up of transmembrane proteins, which conduct surveillance from the cell surface, as well as cellular compartments such as the endosome or ER, constantly scanning the extracellular environment for PAMPs that can be derived from a wide range of microorganisms, including viruses and bacteria. The expression of TLRs appear to be cell-specific and is mainly found in antigen-presenting cells such as dendritic cells (DCs), monocytes and macrophages, as well as on B cells [62]. TLRs recognize a wide variety of PAMPs, and the recognition of these ligands can be converted into specific intracellular responses through the direct interaction of the TLR toll-interleukin 1 receptor (TIR) domain with one of its cytoplasmic TIR-containing signaling adaptor molecules, such as myeloid differentiation primary response protein 88 (MyD88) and TIR domain-containing adaptor-inducing IFNβ (TRIF) [63]. Viral recognition by TLRs has been detailed in several reports [64,65]. In particular, the activation of TLR-3, -4, -7, -8, and 9 can also culminate in type I IFN production [65,66]. TLR3, as a general viral sensor, detects mainly through double stranded RNA (dsRNA), a replication intermediate of both DNA and RNA viruses; TLR4 recognizes envelope proteins from viruses such as mouse mammary tumor virus (MMTV); TLR7 and TLR8 have been identified to recognize ssRNA viruses like influenza and vesicular stomatitis virus (VSV); TLR9 detects dsDNA viruses such as herpesviruses [63]. 

NLRs are stimulated by microbial agonists and collaborate with TLRs to evoke intracellular immune responses through MAPK and caspase signaling cascades upon sensing bacterial components [67]. NLRs are also known to sense both PAMPS from infectious agents and DAMPs (danger‑associated molecular patterns) that arise as a result of insult or injury to the cell, as well as those derived from the environment [68]. Although the direct binding of virus-derived PAMPs to NLRs is yet to be reported, structural and functional studies of the C-terminal domain of NLRX1, a mitochondrial member of the NLR family, has highlighted its ability to bind both ssRNA and dsRNA, implying that some NLRs may be capable of binding viral RNA directly as well [69]. As such, the notion of NLR-mediated recognition of coronaviruses is, therefore, probable and could be further investigated.

The RLH family of purely cytoplasmic PRRs is made up of the following: retinoic acid inducible gene-I (RIG-I or DDX58), melanoma differentiation-associated gene-5 (MDA5 or IFIH1), and laboratory of genetics and physiology 2 (LGP2). RIG-I and MDA5 are PRRs with two N-terminal caspase-recruitment domains (CARDs) followed by a DExD/H box RNA helicase domain; LGP2 lacks the signaling caspase recruitment domains but shares a helicase domain of similar homology and is thought to serve as a regulator of the former [70]. RIG-I and MDA-5 both sense cytoplasmic dsRNA, which the host recognizes as ‘non-self’, via the N-terminal CARDs [71]. However, the two PRRs each sense distinct PAMPs, depending on the length of viral dsRNA, from different RNA viruses. In addition to long dsRNAs (>2 kb) such as the synthetic dsRNA analogue poly-inosinic poly-cytidylic acid [poly(I:C)], MDA5 also recognizes picornaviruses and noroviruses [72,73]. RIG-I, on the other hand, responds to paramyxoviruses, flaviviruses, orthomyxoviruses and rhabdoviruses [61,74,75]. This is through its recognition of a variety of ligands such as relatively short dsRNA (19-mer to 1 kb) and ssRNA (single stranded RNA), both preferably in the presence of a 5'-triphosphate end, full-length RNA viral genomes, the presence of secondary structures such as poly-uridine motifs within 5'-triphosphate genome termini or longer RNA sequences without 5'-triphosphates, such as 3' untranslated regions (UTRs) of the genome [76,77,78,79,80]. 

While the elicitation of TLRs and RLRs by PAMPs trigger their distinct signaling cascades through divergent downstream effectors at varying efficacies, they ultimately cross paths at the juncture of transcriptional activation of interferon regulatory factor 3 (IRF3) [81], IRF7 [82] and nuclear factor kappa-light-chain-enhancer of activated B cells (NF-κB) [83,84,85,86], all of which translocate to the nucleus and activate the transcription of both type I interferons (IFNα and IFNβ) and inflammatory cytokines that eventually culminates in the concerted induction and development of adaptive antiviral immune response.

### 3.4. Type I Interferon Response

The expression and induction of interferons from the cells occurs in response to viral insults and tumor growth [87]. Type I interferons, the major group of cytokines in innate anti-virus defense, bind a specific cell surface heteromeric receptor, the interferon-α/β receptor (IFNAR), which composed of two subunits, IFNAR1 and IFNAR2 [88]. The best characterized type I IFNs can be classified into two major groups, the immediate-early genes (such as IFNβ) that are triggered by the initial response to virus infection, and the delayed-set (such as IFNα subtypes) that rely on a secondary *de novo* protein synthesis pathway [89]. IFN expression is regulated by IFN regulatory factors. In particular, IRF3 and IRF7 play vital roles in activating innate immune response through their respective antiviral response [66]. 

IRF3 has been functionally characterized to consist of a nuclear export signal (NES), a DNA‑binding domain (DBD), a C-terminal IRF association domain (IAD), as well as a number of phosphorylation sites as well as two auto-inhibitory domains that prevents a constitutive activation of the NES, DBD and IAD [90]. Normally found in an inactive cytoplasmic form, IRF3 is phosphorylated as a consequence of virus infection. This activation signal exposes the DBD and IAD and results in the dimerization of IRF3, either as a homodimer or as a heterodimer with IRF7, allowing the activated IRF3 to form a complex with the transcriptional co-activator cAMP-response element-binding protein (CREBP) to translocate to the nucleus and bind to DNA to trigger the transcription and expression of immediate-early IFNs, which signals the JAK-STAT (Janus kinase-Signal Transducer and Activator of Transcription) pathway through the binding of the IFNAR [90]. This leads to the formation of a STAT1-STAT2 heterodimer that teams up with the interferon regulatory DNA binding factor IRF9, which together constitute an activated heterotrimeric factor, the interferon-stimulated gene transcription factor ISGF3, that, through the recognition and binding of specific ISREs, induces downstream expression of innate immunity genes for host defense against virus invasion [91]. 

In contrast to the constitutive expression of IRF3, IRF7 is involved in the positive feedback regulation of IFN production [89]. Expressed only in minute amounts, the induction of IRF7, via virus‑induced activation of ISGF3, results in either homo-dimerization or hetero-dimerization with IRF3 and is subsequently followed by nuclear translocation for the activation of both IFNα and IFNβ genes [92,93]. 

### 3.5. The Effect of Coronaviruses and Arteriviruses on Host Innate Immune Responses

**Figure 2:**
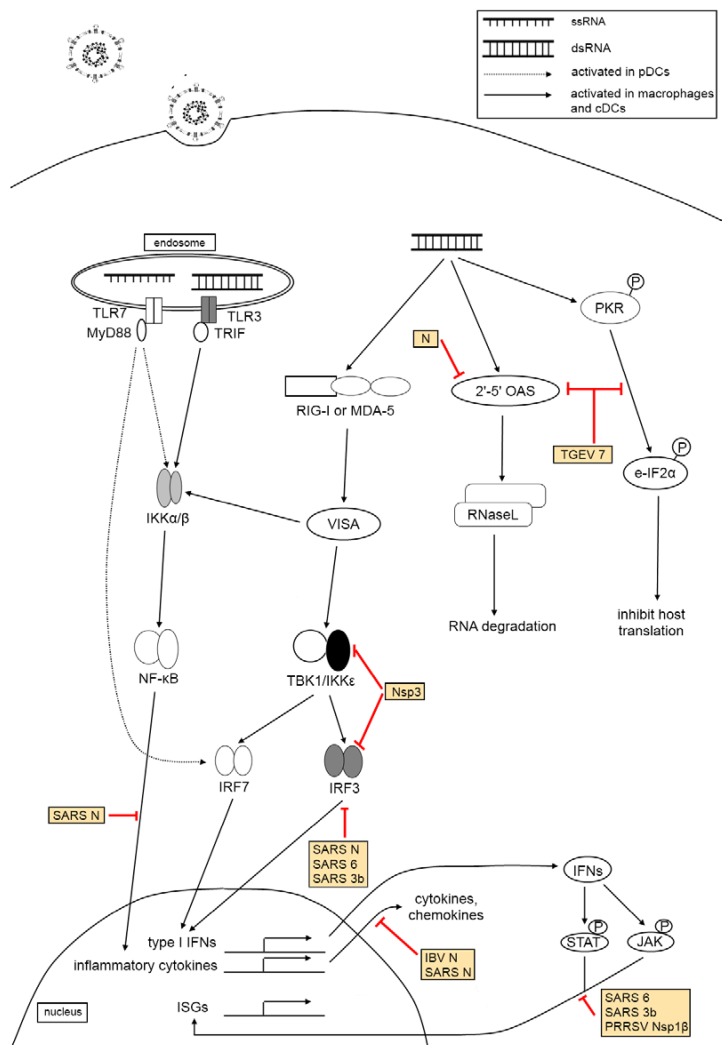
Virus-host interactions in innate immune response. The activation of TLRs-3 and/or -7 as well as cytoplasmic helicases RIG-I and/or MDA-5 triggers signaling pathways resulting in the synthesis of type I IFNs, inflammatory cytokines and ISGs which acts in concert to establish an antiviral state. The activation of both 2'-5' OAS and PKR results in global degradation of cellular RNA and inhibition of translation, which may inhibit viral propagation. Coronaviruses encode many proteins (see yellow boxes) that target multiple steps in the innate immune response mounted by the host cells, ensuring its successful replication in the host.

Coronaviruses and arteriviruses have evolved multiple strategies to avoid elimination from the host. These tactics range from the prevention of detection to inhibition of antiviral responses mounted by the host immune system. All these activities involve virus-host interactions at different levels (Figure 2).

The modulation of SARS-CoV pathogenesis, for example, was reported to be independent of all three types (Types I, II and III) of interferon signaling mechanisms, with SARS-CoV ORF 3b, ORF 6, and nucleocapsid proteins identified to interfere with interferon signaling through various mechanisms [94]. However, STAT1 has been shown by the same group to be crucial in activating innate immune signaling pathways during SARS-CoV infection, with a secondary role in the prevention of uncontrolled cell reproduction [95]. In contrast, PRRSV appears to be receptive to both IFN-α (porcine IFN-α, or Ad5-pIFN-α) and -β (recombinant swine beta interferon, or swIFN-beta) dose-dependent treatment [96,97]; however, pigs infected with PRRSV do not evoke significant IFN responses, with little or no IFN-α and IFN-β production [98,99]. Upstream signaling pathways that may potentially lead to the inhibition of IRF3 activation include the interference of PRRSV with RIG-I signaling events through inactivation of the RIG-I downstream signaling adaptor, MAVS (mitochondrial antiviral signaling protein) [100]. Recent studies have also suggested PRRSV infection inhibits type I IFN production and signaling processes through the impairment of STAT1/STAT2 nuclear translocation [101]. 

One of the most indispensible adaptor proteins in the RLH signaling pathway is MA*VS.* Otherwise known as virus-induced signaling adaptor (VISA), interferon β (IFNβ) promoter stimulator-1 (IPS-1) or CARD-adaptor inducing IFN-β (Cardif), it was discovered independently by four different research groups in 2005 [102,103,104,105], and contains an N-terminal CARD-like domain, which interacts with the CARD domains of RIG-I and MDA5, and a C-terminal transmembrane (TM) region that lodges the protein in the mitochondrial membrane, thus pioneering the connection between mitochondria and innate immunity. A deficiency in, or the cleavage of, MAVS from the mitochondrial membrane results in an arrested antiviral immune response, highlighting the pivotal role MAVS plays in mitochondria-mediated innate antiviral immunity [106]. MAVS can also induce apoptosis independently of Type I IFN activation, with SARS-CoV non-structural protein 15 identified as an inhibitor of MAVS-induced apoptosis to escape host antiviral immune responses [107].

Coronaviruses have devised a number of cell type-specific strategies to inhibit type I IFN responses. One such strategy that these viruses have adopted is to avoid detection of its newly synthesized mRNAs in the cytoplasm through the encoding of a 2'-*O*-methylase (non-structural protein nsp16) that creates a 5'-cap structure analogous to the cellular mRNAs on their mRNAs, thereby escaping detection by Mda5 [108,109]. More importantly, this also highlights the significance of mRNA cap alterations, as such 2'-*O*-methylation, as pertinent molecular signals for the discernment between self and non-self mRNA [108]. However, during the course of viral transcription and replication, uncapped double-stranded RNA intermediates are generated and these may serve as ligands for the RIG-I and/or Mda5. Mouse hepatitis virus (MHV) infection was first reported to activate type I IFN responses in various cell types such as macrophages and microglia in the brains of infected animals, and the virus has also been shown in the same report to be recognized specifically by MDA5 in mouse macrophages [110]. In other reports, both RIG-I and Mda5 have been implicated in the detection of mouse hepatitis virus (MHV) infection, particularly in mouse oligodendrocytes [111], although conflicting evidence has been reported as well, especially since it is not understood how cellular helicases could bind viral replicative/transcriptive intermediates when the latter should have been isolated by the double membrane vesicles [112]. MHV infection can also delay IFNβ-activated ISG induction; however this phenomenon is limited to certain cell types and is observed only when the infection occurs before IFNβ exposure [113]. As such, the general sensing of coronaviruses appears to be cell-type specific, with Type I IFNs derived from plasmacytoid dendritic cells (pDCs), conventional DCs and macrophages being particularly essential in curbing the pathogenesis of mouse coronavirus infection [114].

Detection by the PRRs would activate signaling cascades leading to the production of type I IFNs, resulting in the establishment of an antiviral state. Thus, coronaviruses do not just avoid detection by the host immune system, which appears to be the main strategy of ensuring successful replication [115]; some viruses also encode proteins that function to disrupt the downstream signaling cascades at various points, preventing the establishment of an effective antiviral state when detection of the viral PAMPs has occurred. Indeed, the N protein has also been shown to interfere with the 2', 5'-oligoadenylate synthetase/RNaseL (2'-5'OAS) activation that occurs downstream of IFN induction and which leads to the inhibition of global translation shutdown [116]. This activity is in addition to its suppression of IFNβ induction through the binding of viral RNAs that prevents their detection [117]. 

In addition to the processing of polyproteins, the papain-like proteinase 2 (PLP2) domain of nsp3 has been shown to possess de-ubiquitinating activity [118,119,120]. PLP2 of nsp3 de-ubiquitinates TANK‑binding kinase 1 (TBK1), the activating kinase for IRF3 and IRF3 itself, and sequesters the hypo-phosphorylated TBK1-IRF3 complex in the cytoplasm as well. This prevents IRF3 nuclear translocation [121,122], thereby inhibiting the transcription of type I interferons [123].

*In vitro* activation of chicken splenocytes and peripheral blood leukocytes with IBV has also resulted in an increase in chicken interferon gamma (chIFN-γ) production as a form of cell-mediated immune response [124]. This appears to be a polyclonal, non-specific stimulation as chIFN-γ production levels are also elevated in IBV-stimulated chicken splenocytes which lack prior exposure to IBV, when compared to control un-stimulated cells, as well as in cells exposed to inactivated IBV [124].

Such ineffectual host innate responses could lead to poor cellular responses, which may result in a delay in pathogen clearance and persistent viral infection in compromised pigs, which would present significant advantages to the virus for the subsequent release of viral progeny and spread. 

## 4. The Effect of Virus Infection on Other Host Innate Defenses

### 4.1. Animal Coronavirus Infection and Unfolded Protein Response

The onset of coronavirus-induced apoptosis is intricately linked with other host antiviral innate defenses. Infections with viruses often result in cell cycle arrests and the activation of unfolded protein response (UPR) due to ER stress, both of which may be accompanied by the parallel activation of apoptosis in the infected cells.

During virus replication, newly translated viral proteins accumulate in the ER, which may also cause stress that leads to activation of the UPR. MHV S protein has been shown to activate three UPR transducers, inositol-requiring enzyme 1 (IRE1), activating transcription factor 6 (ATF6) and protein kinase RNA-like endoplasmic reticulum kinase (PERK) [125]. In particular, PERK activation leads to the activation of p38 MAPK [126], which stimulates virus replication [127,128]. SARS-CoV, on the other hand, has been shown to attenuate the IRE1 signaling pathway, which generally led to the down‑regulation of stress responses and UPR, and further decreased apoptotic signaling in infected cells as well, to the likely benefit of the virus for better viral progeny production and release [129].

### 4.2. Virus Infection and the MAPK Signaling Pathway

Coronavirus-induced apoptosis during the late stages of infection is also partially regulated by the p38 MAPK signaling pathway, which in turn up-regulates production of pro-inflammatory cytokines, interleukin (IL)-6 and IL-8, in infected host cells and mount an immune response against virus infection in these cells [130,131]. 

However, while pro-inflammatory cytokines are up-regulated at the transcriptional level during coronavirus infection, there is minimal to moderate up-regulation at the translational level, leading to the hypothesis that the interaction between IBV spike (S) protein and host eukaryotic initiation factor 3 (eIF3) modulates host gene expression, especially genes involved in innate immunity that are activated during coronavirus infection [132]. This also agrees with the hypothesis that, notwithstanding the presence of virus-induced ER stress responses, a significant decline in host mRNA translation in infected cells impedes the expression of ER stress proteins despite elevated mRNA concentrations of the former [133]. Moreover, the negative modulation of p38 MAPK occurs through an up-regulation of the dual-specificity phosphatases 1 (DUSP1) feedback loop during IBV infection, which reduces cytokine production by dephosphorylation of phosphor-threonine and phosphor-tyrosine residues on activated p38 MAPKs [130].

PRRSV infection of porcine macrophages, too, appears to be under the control of the MAPK/ERK pathway, through various signaling pathways activated by the latter that regulate a variety of cellular processes [134]. In particular, chemical inhibition of the ERK pathway resulted in an attenuation of PRRSV infection during the early stages of infection post-virus attachment, during which a notable reduction in viral sub-genomic mRNA synthesis and translation, as well as in progeny virus release, was observed [134]. An increase in IL-10 production during the early stages of infection has also been observed in pigs infected with PRRSV. The up-regulation of IL-10, an important cytokine in the attenuation of innate and adaptive immune responses, is advantageous for PRRSV to sustain for a longer period of time in the host, and the PRRSV N protein has been suggested as the viral protein responsible for mediating IL-10 induction [135].

### 4.3. Induction of Other Pro-Inflammatory Cytokines

Likewise, the expression of pro-inflammatory mediators, IL-1β, IL-6, IL-8, and TNF-α, are up‑regulated during EAV infection of equine endothelial cells and macrophages, with virulent EAV strains activating sufficiently greater amounts of these cytokines, especially TNF-α, than avirulent EAV strains [136]. Moreover, the expression of pro-inflammatory cytokines, IFN-α, TNF-α, IL-1 and IL-6, is also up-regulated during porcine respiratory coronavirus and TGEV infection, and the differential modulation of which are postulated to function as crucial mediators of viral respiratory diseases in pigs [41]. In particular, early type I IFN production in coronavirus-infected pigs effectuated immunomodulatory responses, which suggests the potential ability of IFN inducers as an effective antiviral strategy to control the rate of coronavirus infections in swine populations [137].

RANTES (regulated upon activation, normal T cell expressed and secreted) is a known member of the pro-inflammatory CC chemokines family that function in the modulation of the migration of inflammatory cells such as monocytes and Natural Killer cells to sites of infection, particularly as a form of host antiviral immune response during virus infection [138]. Like coronaviruses, PRRSV infection also triggers the activation of pro-inflammatory cytokines and chemokines, including RANTES [139], which may be fundamental in initiating the pathological conditions associated with the arterivirus. The activation of RANTES transcription also requires the participation of adaptor molecules from the TLR signaling pathway, such as MyD88, TRIF and TNF receptor-associated factor 6 (TRAF6) [139].

Interestingly, respiratory PRRSV/PRCV viral co-infections that commonly occurs in pigs, on the other hand, frequently leads to severely attenuated innate and adaptive immune responses in order to extend the pathogenicity of PRRSV—thus down-regulating innate immunity—and renders the host more vulnerable to subsequent infections by PRCV, or other respiratory viruses, that instead up‑regulates innate immunity, possibly due to further, more severe damage to pulmonary cells and tissue and which may ultimately lead to a more critical form of pneumonia [140].

### 4.4. Coronaviruses and Autophagy

Other types of host innate defense against virus infection include processes such as autophagy, a vital physiological process in which cells degrade their own organelles in response to a severe lack of nutrients or other cellular stresses, so as to facilitate the removal of damaged cellular components and prevent the build-up of unwanted products in the cells [141]. Autophagy is also known to mount crucial innate immune responses against invading pathogens, with degradation of the latter through autophagy; on the other hand, autophagosomes may instead help promote virus infection by bringing together viral replicase proteins [142]. Multiple host-derived cytokines have emerged to play differential roles in regulating the onset of this essential mechanism as well. Of note are the T-helper cells (Th1) group of cytokines, including IFN-γ, TNF-α, IL-1, IL-2 and IL-6, which have the ability to induce autophagy, while Th2 cytokines such as IL-4, IL-10 and IL-13 have been shown to inhibit autophagy [143]. 

Recent studies have shown the ability of IBV to induce autophagy during infection independently of cellular stress or nutrient deprivation, and this can be made possible through the functions of IBV nsp 6, as well as in nsp6 orthologs of mammalian coronaviruses such as MHV and arteriviruses such as PRRSV, although a direct link between IBV infection and autophagosome formation could not be established [22]. As such, the induction of autophagy that has previously been reported during coronavirus and arterivirus replication may instead be an example of an innate defense tactic against infection to remove unwanted viral particles, while nsp6—as well as its respective orthologs—may alternatively modify adaptive immune strategies by targeting the breakdown of immunomodulatory proteins synthesized by the ER in autophagosomes [22].

## 5. The Effect of Virus Infection on Cell Cycle Regulation

**Figure 3:**
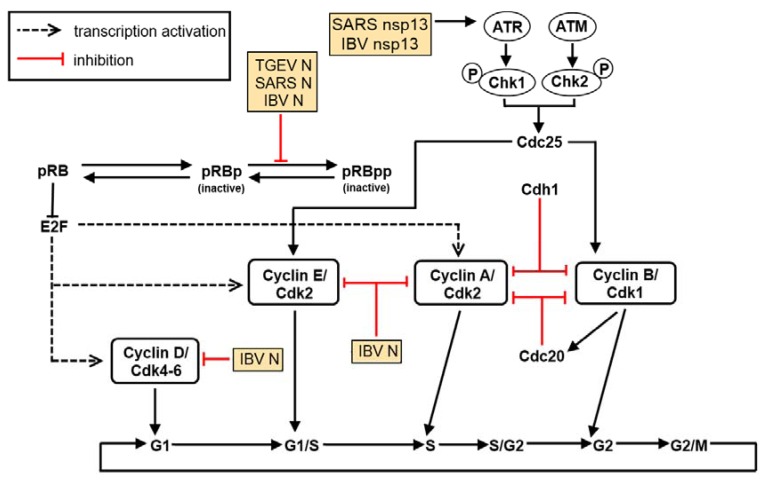
Coronavirus-encoded proteins interfere with the cell cycle. Cell cycle progression is mediated through the temporal expression levels of different cyclins and CDKs. Coronaviruses have been confirmed to cause cell cycle arrest at both G0/G1 and G2/M phases. This arrest is mediated through the nucleocapsid protein (N) as well as other virus-encoded non-structural (replicase) and accessory proteins such as nsp13.

Viruses may also exploit the host cell cycle to benefit their own replication [144] (Figure 3). Cell cycle regulation typically involves mechanisms critical to cell sustainability, such as the surveillance and correction of genetic damage and the impediment of unrestrained cell division. Cyclins and cyclin‑dependent kinases (CDKs) are well-known regulatory proteins, and the activation of which will dictate a cell’s progress through the cell cycle. Briefly, activated heterodimers consisting of both cyclins and CDKs as the regulatory and catalytic subunits, respectively, will alter the phosphorylation state of various target proteins for progression into the next stage of the cell cycle. CDK inhibitors include tumor suppressors such as the *cip/kip* (*CDK interacting protein/Kinase inhibitory protein*) and the INK4a/ARF (*In*hibitor of *K*inase 4/*A*lternative *R*eading *F*rame) family of genes that thwart progression to the next stage of the cell cycle [145].

The inactivation of CDKs can also be a reversible process through the phosphorylation of essential residues Tyr 15 and Thr 14, both of which are located within the CDK ATP-binding loop [146]. Dual‑specificity phosphatases of the Cdc25 (cell division cycle 25) family are responsible for the dephosphorylation of Tyr 15 and Thr 14 [147]. These Cdc25 proteins are, in turn, inactivated by Chk1/Chk2-mediated phosphorylation; this inactivation averts Cdk dephosphorylation and impedes its subsequent activation [148]. Chk1/Chk2 activation after DNA damage and/or DNA replication hindrance is dependent on the ataxia-telangiectasia mutated (ATM) and ATM/Rad3-related (ATR) protein kinases in mammalian cells [149,150].

IBV infection of cultured cells results in cell cycle arrest, at both S and G2/M phases, to boost viral replication and to enhance the production of viral proteins as well. This p53-independent growth inhibitory outcome is catalyzed by regulation of the expression of multiple cell cycle regulatory genes such as corresponding CDK complexes [50] and the down-regulation of down-regulation of G1 phase regulatory cyclins D1 and D2 [151], as well as through systemic modulation of ATR-dependent cellular DNA damage response [152]. Specifically, the interaction of coronavirus nsp13 with the p125 subunit of DNA polymerase δ was discovered to trigger DNA replication stress in cells during IBV infection, which eventually led to cell cycle arrest at the S phase [152].

It has also been observed that the N protein of several coronaviruses can localize in the nucleolus where it may perturb cell cycle activities of the host cell for the benefit of viral mRNA synthesis [153,154,155,156]. IBV N, for example, appears to target CDK2, cyclins A and D1 for proteasome-mediated degradation [50,157] and cause the accumulation of hypophosphorylated retinoblastoma (pRB), resulting in the downregulation of CDK1, cyclins E and B1 [50]. 

## 6. The Effect of Virus Infection on the Host Translation Machinery

The regulation of host protein synthesis, especially at the initiation stage, is often a regular viral objective for the extensive reduction of host protein translation so as to construct the most favorable condition for viral replication and progeny assembly. Protein kinase R (PKR) is a prevalent serine/threonine protein kinase that can be induced by interferon in its latent state, and whose activation is dependent by the presence of dsRNA products, is one such viral target. PKR plays an important role in the cellular anti-viral response pathway, and becomes activated via auto-phosphorylation upon binding to virus-derived dsRNA, which then subsequently leads to host translation inhibition through phosphorylation of the alpha subunit of eukaryotic initiation factor 2, eIF2alpha [158]. 

The dephosphorylation of eIF2alpha is established by cellular protein phosphatase-1 (PP1), which functions to regular various cellular processes through the physical interaction of its catalytic subunit (PP1c) with regulatory proteins such as GADD34/CHOP that is induced by DNA damage signals [159,160]. 

In the case of IBV, for example, eIF2alpha phosphorylation was significantly suppressed in both human and animal cells during infection. The dephosphorylation of eIF2alpha during IBV infection appears to be induced by the up-regulation of GADD34 expression, which, together with the simultaneous attenuation of PKR auto-phosphorylation following IBV infection in these cells, serves as a viral regulatory tactic in boosting coronavirus replication while managing the delicate balance of *de novo* protein translation along with the identification of IBV nsp2 as a potential, albeit weak, mediator in the dose-dependent inhibition of PKR activation [161]. TGEV, too, suppresses both cellular RNA degradation and eIF2a phosphorylation during infection through an interaction between TGEV protein 7 and PP1 that regulates host antiviral responses and extends the period of viral progeny dispersal as well [53]. 

A subunit of the eukaryotic initiation factor 3, eIF3f, has also been discovered to modulate host translation inhibitory effects through physical interaction with coronavirus spike protein [132]. As this inhibition takes place during the late stages of the coronavirus replication cycle, translation of virus‑induced transcripts are largely affected, especially pro-inflammatory cytokines and chemokines. This could account for the fact that while IL-6 mRNA expression is up-regulated during the early stages of coronavirus infection, insubstantial increase in IL-6 protein expression was observed [132]. The inhibition of host protein synthesis through the interaction between coronavirus spike protein and eIF3f may therefore have a significant impact on the modulation of coronavirus pathogenicity.

## 7. Cellular Factors Involved in the Virus Life Cycle

**Figure 4:**
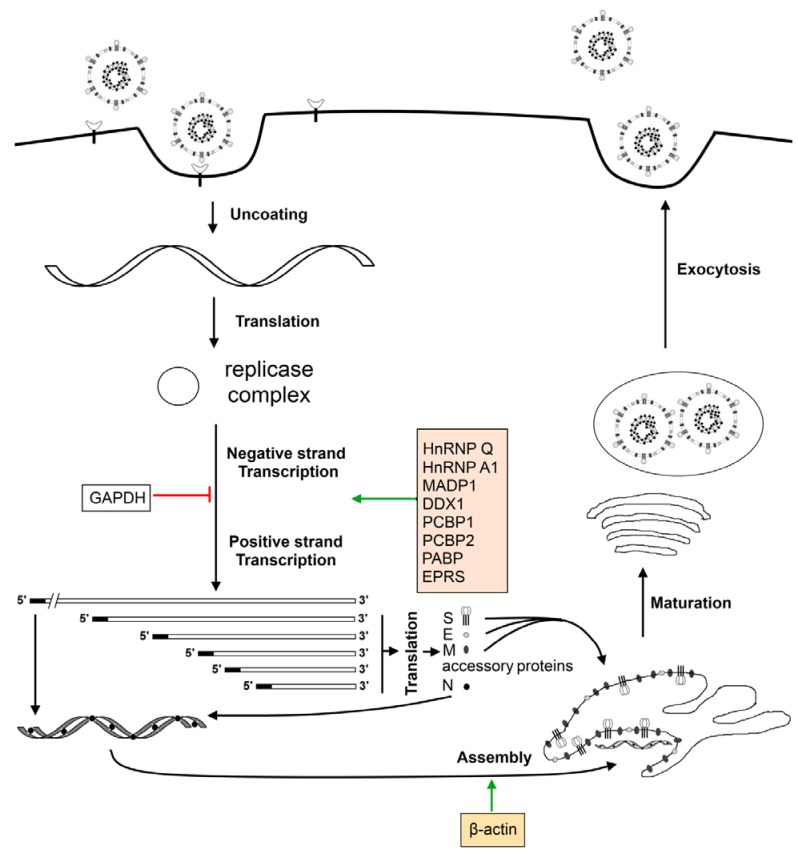
Host proteins and the coronavirus life cycle. Virus particle attaches onto the host cell via cellular receptors on the surface and enters. Entry is followed by the uncoating of the ribonucleocapsid to expose the positive-sense genomic RNA which is translated by the host ribosomes to yield the viral replication complex. The viral replication complex continues with viral transcription and genome replication and, with the aid of host proteins such as hnRNPA1, yields a nested set of positive-sense sub-genomic sized mRNAs as well as the full-length virus genome. Sub-genomic sized mRNAs are translated by host ribosomes into viral structural (S, E, M, N) and accessory proteins. The N protein packages the positive-sense genomic RNA into a ribonucleocapsid and is assembled into the virus particles with the help of β-actin. The newly formed virus particles undergo maturation when passing through the Golgi and exit the host cell via exocytosis.

Host proteins are also known to play a role in the virus life cycle, especially during viral RNA replication and transcription (Figure 4). The most well studied host protein that interacts with the coronavirus genome is heterogeneous nuclear ribonucleoprotein A1 (hnRNP A1), a nuclear protein, whose biological function is to regulate alternative splicing of cellular RNAs [162,163]. The hnRNP A1 has been shown to bind to both negative-sense leader sequence and negative-sense intergenic (IG) sequence of MHV [164]. The formation of a RNP complex among hnRNP A1, negative-sense leader and IG sequences has also been demonstrated [165]. In addition to its ability to interact with the coronavirus RNA, hnRNP A1 was also found to interact and co-localize with N protein [166,167], an important player in coronavirus RNA synthesis [168,169]. It has also been highlighted that hnRNP A1 may be required to recruit other cellular proteins to the replicase complex [170]. 

To ascertain the involvement of cellular factors in TGEV RNA synthesis, TGEV 3' and 5' genome ends were used as baits for RNA affinity protein purification [171]. Of the ten cellular proteins pulled down with either genome end, poly(A)-binding protein (PABP), hnRNP Q, and glutamyl-prolyl-tRNA synthetase (EPRS) were confirmed to enhance TGEV infection through their respective interactions with the TGEV 3' end, while glyceraldehyde 3-phosphate dehydrogenase (GAPDH)—originally employed as a control—was discovered, surprisingly, to have a diminishing effect on TGEV infection instead [171].

For arteriviruses, the common leader sequence of EAV sub-genomic viral RNAs, too, possesses the ability to interact several cellular proteins from the cytoplasmic fractions of Vero cells, likely for the modulation of EAV RNA synthesis [172]. 

Recent reports, based on a yeast-based three-hybrid system to identify RNA—RNA-binding regulatory protein interactions [173], and using the 5'-UTR (untranslated region) of SARS coronavirus as bait, identified zinc finger CCHC-type and RNA-binding motif 1 (MADP1) as a potential cellular protein that interacts with SARS-CoV, and this was eventually established via an *in vitro* pull-down assay with the 5'-UTR of IBV [174]. MADP1 was also shown by the same authors to play a role in the early stages of the coronavirus replication cycle, with the RNA recognition motif in the N-terminal region of the protein interacting with stem loop 1 of the IBV 5'-UTR. Specifically, this protein translocates from the nucleus to the cytoplasm of the cell during IBV infection and co-localizes, in part, with viral replicase/transcriptase complexes (RTCs) in order to enhance viral replication and coronavirus RNA synthesis [174]. 

Interaction with viral RNA is not the only way by which cellular proteins can take part in viral RNA synthesis; protein-protein interactions with the viral replicase complex can modulate this process as well. This was confirmed in coronaviruses with a yeast two-hybrid screen that was performed using IBV nsp14 as a bait protein, which ultimately led to the discovery of DDX1, an ATP-dependent RNA helicase in the DExD/H helicase family, as an interacting partner that translocates from the nucleus to the cytoplasm and enhances IBV replication in cultured mammalian cells through subcellular colocalization with nsp14, an exonuclease, during infection [175]. 

Another yeast two-hybrid screen, with IBV membrane (M) protein as the bait, identified beta-actin as an interacting partner, leading to the suggestion that actin filaments may possess the ability to participate in virion assembly and budding during the coronavirus replication cycle [176]. This interaction may possibly lead to the incorporation of actin into the mature virion. In fact, recent proteomic analysis of purified IBV particles through two-dimensional gel electrophoresis and subsequent mass spectrometry have confirmed, among others, an abundance of actin within the virus particles, further cementing the hypothesis that cytoskeletal elements play crucial roles in IBV replication [177].

Retinoblastoma tumor suppressor proteins are also interacting partners of coronavirus nsp15, an endoribonuclease, which may result in alterations to the cell cycle that impact coronavirus infection and viral progeny release [178]. Similarly, with nsp1β, the functional proteolytic product of PRRSV nsp1, as a bait protein, cellular poly(C)-binding proteins 1 and 2 (PCBP1 and PCBP2, respectively) have been identified interacting partners of the former, with specific functions in modulating PRRSV replication and RNA synthesis [179]. 

## 8. Conclusions

The relationship between a virus and its host is complex; the virus must evolve evasive strategies to avoid detection and immunological defense mechanisms from the host while the host must develop various lines of defense in order to combat viral invasion. 

As highlighted in this review, the diverse virus-host interactions established during animal coronavirus and arterivirus infections may have direct implications on viral replication itself, or they may also lead to the modification of numerous signaling pathways, such as cellular stress and/or host antiviral innate immune response, as a means to expedite viral replication and pathogenesis.

The intricate network between a virus and its host is a complicated affair that involves many players—derived from both virus and host—which are pitted against one another in the battle for ascendancy; certain host processes are readily assimilated and manipulated by the virus in its bid to impede host antiviral responses while the host is itself armored with several lines of defense mechanisms and numerous antiviral factors to combat viral invasion to prevent its spread. 

While much progress has been made in the elucidation of the various regulatory pathways that, under the most propitious of conditions, will allow virus and host to co-exist in an uneasy truce, a clearer understanding of the complex interplay between the virus and its host could give new insights into the role of main players, such as those that govern the onset of apoptosis, or type I interferons and their regulators, and lead to the discovery of novel and/or universal targets for the therapeutic mediation against pathogenic infection. 
